# Tuft cell-mediated pathogen sensing and inflammation resolution

**DOI:** 10.1042/BST20253057

**Published:** 2025-11-25

**Authors:** Liquan Huang, Yuan-Yuan Peng, Defu Yu

**Affiliations:** College of Life Sciences, Zhejiang University, Hangzhou, Zhejiang, 310058, China

**Keywords:** host–pathogen interactions, immune response, innate immunity, stem cells, taste

## Abstract

Tuft cells are a rare type of epithelial cells characterized not only by their tuft-like structure but also by the expression of specific genes, including those encoding the transcription factor Pou2f3 and canonical gustatory signaling proteins. However, tuft cells can be heterogeneous in various features, both across different tissues and within the same tissue. Homeostatic tuft cells are generated from stem/progenitor cells; however, their formation and gene expression profiles are regulated epigenetically and in response to changes in their microenvironments. Ectopic formation of tuft cells, their transdifferentiation into other cell types, and dedifferentiation to stem/progenitor cells have also been found in some tissues upon severe injuries. Tuft cells function as chemosensory sentinels and can detect a variety of pathogens such as bacteria, protists, and helminths with their cell surface receptors. Activation of these receptors in turn activates intracellular signaling cascades, leading to the release of output effectors: the cytokine IL-25, the eicosanoids, and the transmitters acetyl choline and ATP, some of which act on group 2 innate lymphoid cells, triggering innate immune responses, or on neighboring epithelial cells to accelerate cilia beating and increase mucus secretion, or on the nerve terminals to initiate neuroimmune responses. Some tuft cells are also critical to inflammation resolution and tissue repair—an important part of the healing and recovery process. Further elucidation of tuft cells’ ligands, respective receptors and downstream signaling pathways, and output effectors can provide more insights into these cells’ pivotal roles in health and disease.

## Introduction

Tuft cells with a characteristic morphology of tuft-shaped protrusions at the apical end were first reported nearly 70 years ago [1,2]. Their functions, however, remained elusive until recently [3–5]. These cells apparently play important roles as chemosensory sentinels and modulate the host’s defense mechanisms against noxious stimuli and pathogens such as allergens, bacteria, protists, and parasitic helminths, and at the same time, protect the host tissues and organs from further damage by participating in inflammation resolution and wound repair. While much research progress has been made on tuft cell functions in the past few years, further studies are needed to comprehensively understand the heterogeneity of these cells, their epigenetic regulation, their ability to dedifferentiate and transdifferentiate, their distinct functions in different tissues as well as their pivotal contributions to our physiology and health.

### Tuft cell heterogeneity

**Figure 1:**
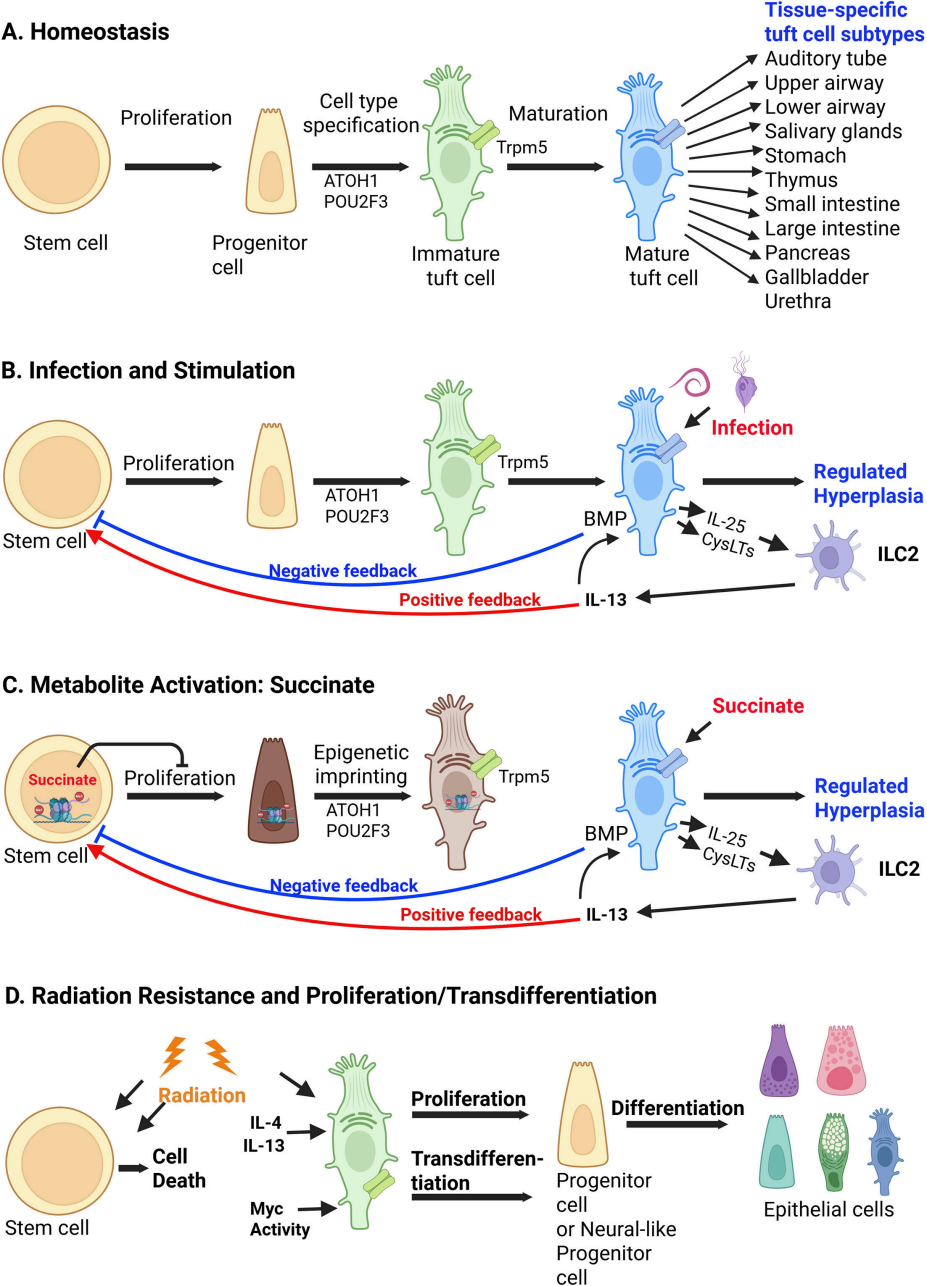
Tuft cell formation. (**A**) Homeostatic tuft cells are generated from stem/progenitor cells and regulated by the transcription factors ATOH1 and POU2F3. Tuft cells in different tissues express a distinct set of genes. (**B**) Upon infection or stimulation, tuft cells undergo hyperplasia by a positive feedback loop, which is counteracted by a negative one. (**C**) The metabolite succinate can act on its receptor on tuft cells and activate the tuft cell-ILC2-stem cell circuit while the intracellular succinate can trigger epigenetic modification, inhibiting the stem cell proliferation and generating imprinted tuft cells with compromised functions. (**D**) Radiation can ablate stem cells and other cells except tuft cells, which proliferate and differentiate into all other types of epithelial cells whereas Myc activity induces transdifferentiation of tuft cells into neural-like progenitor cells. Created with BioRender.com.

Tuft cells have been found in many tissues and organs, including the salivary glands, small and large intestines, stomach, pancreas, nasal cavity, upper and lower airways, auditory tube, thymus, gallbladder, and urethra [6–11]. These different tissues provide distinct microenvironments, to which tuft cells adapt and develop specialized functions in immune defense and subsequent recovery in accordance with their special circumstances (Figure 1A). Consequently, tuft cells exhibit heterogeneity across different tissues and even within the same tissue. Nevertheless, several common features enable their identification across tissues [12]. In addition to their distinct tuft-like morphology, all tuft cells and type II taste bud cells express the transcription factor Pou class 2 homeobox 3 (Pou2f3) and canonical taste signaling proteins such as the transient receptor potential cation channel subfamily M member 5 (Trpm5) [5,13]. Additional molecular markers commonly used to identify these cells include the heterotrimeric G protein α subunit α-gustducin (Gene: *Gnat3*), G protein γ subunit Gγ13 (Gene: *Gng13*), doublecortin-like kinase 1 (DCLK1), hematopoietic prostaglandin D synthase (HPGDS), interleukin-25 (IL-25), sialic acid-binding immunoglobulin-like lectin F (SIGLECF), cyclooxygenase 1 (COX1), COX2, choline acetyltransferase (ChAT), and the lymphoid restricted membrane protein (LRMP) [14]. However, expression of these markers is not universal among tuft cells. For example, murine olfactory tuft cells lack DCLK1 [15], whereas human intestinal tuft cells do not express SIGLECF, IL-25, or DCLK1 [16]. These variations in gene expression profiles underscore the heterogeneity of tuft cells.

Given this heterogeneity, efforts have been made to classify tuft cells into subtypes. Based on gene expression profiles, intestinal tuft cells were initially categorized into two subtypes: tuft-1 and tuft-2 [14]. Tuft-1 cells express neuronal-associated genes such as ChAT (an enzyme responsible for synthesizing the neurotransmitter acetylcholine (ACh)), and tubulin polymerization-promoting protein family member 3 (TPPP3), whereas tuft-2 cells express immune-associated genes, including the marker protein tyrosine phosphatase receptor type C (CD45/PTPRC) and SH2 domain-containing 6 (SH2D6) [17]. More recent studies, however, indicate that tuft-1 and tuft-2 cells are distributed along the crypt–villus axis in the small intestine: tuft-1 cells predominate near crypt base while tuft-2 cells are more abundant near villus tips. This distribution aligns with cellular differentiation and maturation from crypt to villus tip, suggesting that tuft-1 and tuft-2 may represent the same subtype at different developmental stages rather than distinct subtypes [18]. Further research is needed to determine whether tuft cells from various tissues can be classified into subtypes based on maturation stages, selective response to environmental cues, or effector outputs on adjacent cells.

### Tuft cell generation, epigenetic regulation, dedifferentiation, and transdifferentiation

Tuft cells exhibit widely varying lifespans, from a few days to many years [19,20]. Their turnover is regulated by a plethora of factors. During development, the stem/progenitor cells are programmed to express specific transcription factors. ATOH1 is critical to cell fate determination, while Pou2f3, in turn, directs the expression of genes that commits these cells into tuft cells or type II taste bud cells expressing sweet, bitter, and umami receptors along with their downstream signaling proteins (Figure 1A) [21,22].

Under specific conditions, external stimuli can markedly upregulate tuft cell numbers, leading to hyperplasia (Figure 1B). For instance, parasitic helminth infections or gut microbiome perturbations are detected by small intestinal tuft cells, which secrete the effector cytokine IL-25. This cytokine activates group 2 innate lymphoid cells (ILC2s), triggering type 2 innate immune responses and IL-13 production. IL-13 acts on its receptors on intestinal stem cells, promoting the differentiation of additional tuft and goblet cells and resulting in hyperplasia of both cell types [3–5,23,24]. This tuft cell-ILC2 circuit is thus essential for the ‘weep and sweep’ response that expels invading gut pathogens. To calibrate the innate immune response to specific pathogens, a negative feedback regulation mechanism is also operative: IL-13 produced by ILC2 cells binds to its receptor on tuft cells, inducing expression of bone morphogenetic protein (BMP) ligands. These BMPs engage receptors on stem/progenitor cells, suppressing SYR-Box transcription factor 4 (SOX4) expression and thereby further tuft cell generation [25].

The gut microbiota can be altered by allergies, inflammation responses, antibiotic treatments, and various other factors, potentially enriching succinate-producing microbes [23]. Succinate stimulates its receptor Sucnr1 on tuft and stem cells, thereby triggering a tuft cell-mediated type 2 innate immune response and activating histone deacetylase 3 (HDAC3) in intestinal stem cells, respectively (Figure 1C). HDAC3 deacetylates target genes, suppressing their expression; one such gene is *Spry2*, which encodes the tuft cell suppressor sprout RTK signaling antagonist 2. Reduced *Spry2* expression promotes stem cell proliferation and differentiation, leading to tuft and goblet cell hyperplasia [26]. Conversely, colonization by short-chain fatty acid (SCFA) butyrate-producing microbes or direct exposure to butyrate reduces HDAC3 activity and Pou2f3 expression in the ileum, thereby restricting stem cell differentiation into tuft cells, reducing homeostatic tuft cell numbers, and blocking IL-13-induced tuft cell hyperplasia [27]. HDAC3 is also sensitive to feeding schedule, which activates the transforming growth factor-β (TGF-β) signaling pathway, resulting in rhythmic Pou2f3 expression and diurnal tuft cell biogenesis [28].

Furthermore, inflammatory stimuli can alter cellular metabolism in intestinal stem cells, leading to succinate accumulation and subsequent epigenetic reprogramming [29]. This epigenetic modulation impairs intestinal stem cells' regeneration, with inflammatory imprint persisting in differentiated cells, such as tuft and goblet cells, thereby affecting their capacity to detect and respond to subsequent challenges. In organs like the distal lung and pancreas where tuft cells normally are absent, severe injury induces ectopic tuft cell formation in the distal lung [30,31] and pancreatic duct [32,33]. In the distal lung, ectopic tuft cells arise from activated stem cells, whereas in the pancreas, they transdifferentiate from differentiated acinar cells [34,35]. During pancreatic ductal adenocarcinoma progression, metaplastic tuft cells transdifferentiate into neural-like progenitor cells, correlating with poor patient survival (Figure 1D) [36]. Thus, these ectopic tuft cells play critical roles in tumor progression, inflammation resolution, and wound healing [37,38]. Further research into these cells may elucidate novel regulatory mechanisms governing both normal and ectopic tuft cell generation, cell type specification, and physiological functions.

 Traditionally considered post-mitotic, tuft cells in human small intestinal organoids proliferate upon IL-4 and/or IL-13 stimulation and differentiate into all other epithelial cell types (Figure 1D) [39]. Unlike regular intestinal stem cells, these tuft cells are radiation-resistant and capable of regenerating the entire epithelial cell repertoire to repair damaged gut tissue. Consequently, tuft cell heterogeneity arises from genetic and environmental factors. Transcriptional networks, including ATOH1 and Pou2f3, regulate core tuft cell gene expression, while tissue-specific mechanisms confer unique characteristics to tuft cells in different tissues [40,41]. During maturation, tuft cell gene expression profiles may shift to modulate their functions. Extrinsic factors, such as inflammatory signals and microbial metabolites, further modulate gene expression epigenetically, generating imprinted tuft cells with memory, thereby enhancing their diversity [29].

### Tuft cell input signals

**Figure 2:**
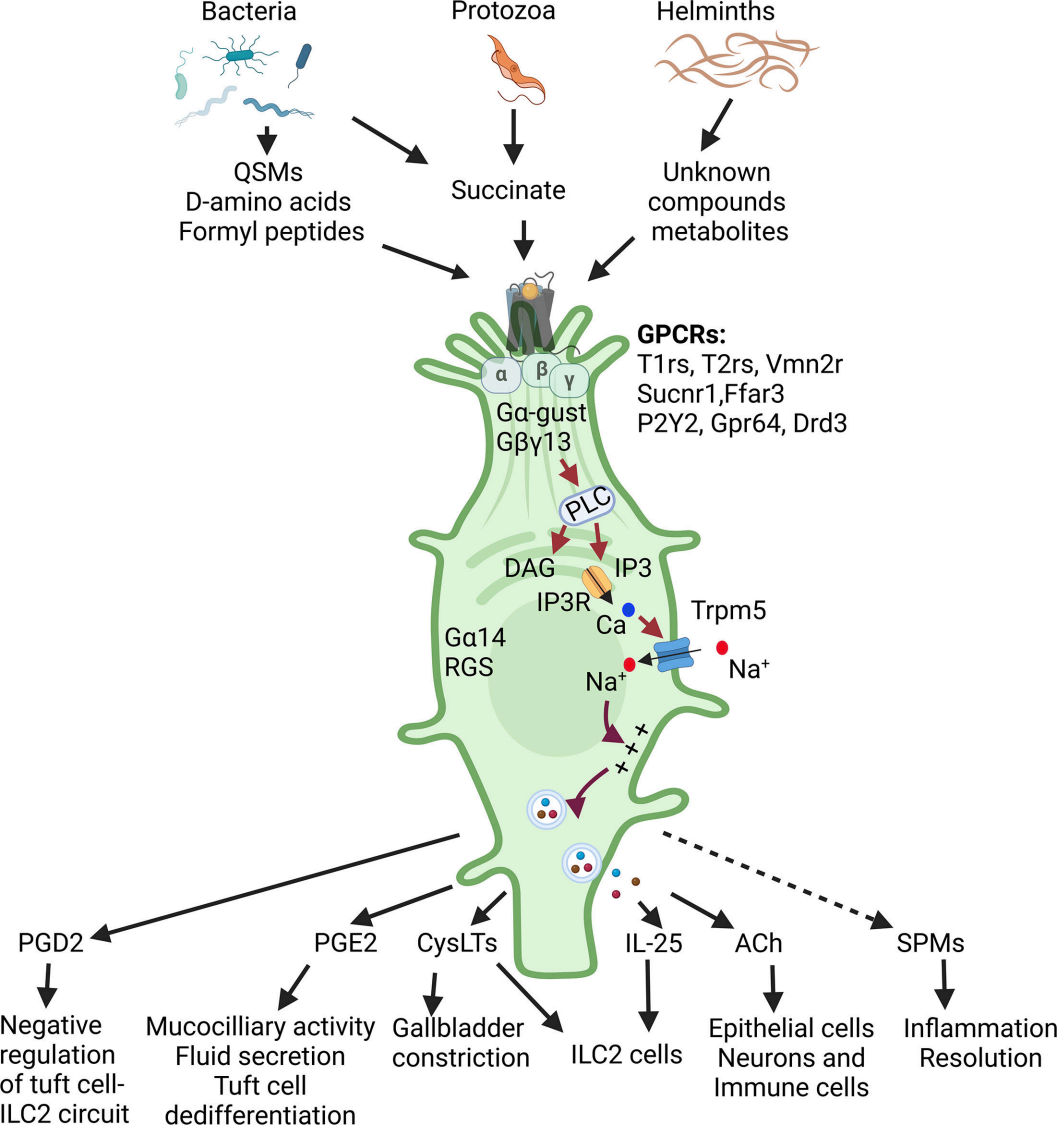
Tuft cells sense environmental cues, initiate signaling cascades, and release output effectors. Bacteria, protozoa, parasitic helminths, and other pathogens produce some substances that are sensed by tuft cells, which activates intracellular signaling networks, leading to the release of one or more types of output effectors. These effectors act on adjacent cells such as other epithelial cells, neurons and immune cells, carrying out the physiological functions. Created with BioRender.com.

Tuft cells detect various environmental cues, with their tissue-specific heterogeneity, resulting in distinct receptor repertoires across subtypes. A number of G protein-coupled receptors (GPCRs) have been identified in tuft cells, including taste receptors such as Tas1r2, Tas1r3 and Tas2r17, Tas2r36, Tas2r143, Tas2r126, and olfactory receptor Vmn2r26 in intestinal and gastric tuft cells [17,42–44]. Bitter-tasting substances, helminth metabolites, or microbial products from *Shigella* and *Ruminococcus gnavus* activate these receptors (Figure 2) [45].

In the upper airway, bacterial quorum-sensing molecules or formyl peptides activate Tas2rs in tuft cells, triggering antimicrobial peptide release from adjacent cells, whereas the sweet taste receptor (Tas1r2/Tas1r3) responds to bacterial D-amino acids, inhibiting Tas2r-mediated signaling and defensin secretion, thus increasing cell death [46,47]. Consequently, bitter and sweet taste receptors exhibit antagonistic functions, reflecting a dynamic interplay between host cells and pathogenic microbes [46,47]. The role of Tas1r3 in intestinal tuft cells remains unclear, as it is insensitive to sweet or umami compounds without Tas1r1 or Tas1r2 [44,48]. However, the possibility that Tas1r3 forms a functional heterodimer with another GPCR, as seen in other Family C GPCRs, cannot be ruled out.

The succinate receptor Sucnr1, the most thoroughly studied receptor in intestinal and tracheal tuft cells, detects succinate produced by certain gut microbes and helminths, thereby triggering type 2 innate immunity and tuft cell hyperplasia [23,49]. Other microbial metabolites, such as short-chain fatty acids propionate and butyrate, are detected by free fatty acid receptor 3 (Ffar3) on tuft cells [50,51]. Another GPCR, P2Y2, expressed in tracheal tuft cells, is activated by ATP released from adjacent cells; activated tuft cells, in turn, release cysteinyl leukotrienes (CysLTs), eliciting immune responses [52]. Additionally, Adgrg2/Gpr64 and dopamine receptor D3 (Drd3) have been identified in tuft cells though their functions remain to be elucidated (Figure 2).

Although lysates or substances from pathogens are known to stimulate tuft cells [53], the corresponding receptors remain unidentified. Conversely, ligands for many of the GPCRs expressed in tuft cells are undetermined, likely produced by pathogens or damaged cells. Matching receptors to their agonists is crucial for classifying tuft cell subtypes, as receptor repertoires and ligand profiles serve as key molecular markers.

### Tuft cell output signals

Upon activation, tuft cells release effector molecules, including the cytokine IL-25, eicosanoids such as prostaglandin D_2_ (PGD_2_) and E_2_ (PGE_2_), CysLTs, and transmitters ACh and ATP (Figure 2).

IL-25 binds to its receptor complex IL-17RA/IL-17RB on ILC2, triggering type 2 innate immune response and IL-13 production, which promotes tuft cell hyperplasia [54]. Additionally, IL-25 acts on tuft cells’ own IL-17RA/IL-17RB, negatively regulating its own release from these tuft cells [55,56].

Tuft cells express enzymes essential for eicosanoid synthesis, including cytosolic phospholipase A_2_ (cPLA_2_, Pla2g4a), which liberates arachidonic acid from plasma membrane phospholipids. This is subsequently metabolized by arachidonate-5-lipoxygenase (ALOX5), leukotriene C_4_ synthase (LTC4S), prostaglandin-endoperoxide synthase 1 and 2 (PTGS1, PTGS2, also known as COX1 and COX2) into CysLTs, PGD_2_, and PGE_2_.

In responses to helminth or *Shigella* infection*,* tuft cells release PGD_2_, which activates the CRTH2 (chemoattractant receptor-homologous molecule expressed on Th2 cells) receptor on small intestinal stem cells and tuft cells, suppressing tuft cell hyperplasia and modulating ILC2-mediated inflammation [17,53,57,58]. In the trachea, tuft cells produce PGE_2_, enhancing fluid secretion and mucociliary function [59]. During colitis, PGE_2_ promotes tuft cell dedifferentiation into stem-like cells, potentially leading to tumorigenesis, which can be inhibited by COX inhibitors [60].

CysLTs are produced by tuft cells in the gut, gallbladder, and airway. In the trachea and small intestine, CysLTs amplify the IL-25-mediated activation of ILC2s, promoting tuft cell hyperplasia [52,61], although this enhancement is dispensable for anti-protozoal responses [62]. In contrast, biliary tuft cell-derived CysLTs induce gallbladder constriction by acting on smooth muscle [63].

Acetylcholine (ACh), a neurotransmitter synthesized by choline acetyltransferase (ChAT) in tuft cells of the intestine, airway, and urethra, does not contribute to type 2 immune responses but directly impairs helminth fitness and egg production in the intestinal lumen or induce chloride secretion from epithelial cells, eliminating luminal helminths [64,65]. In addition, ACh mediates intestinal contraction in ‘weep and sweep’ responses, enhances ciliary beating and induces breathing pause in the trachea, and stimulates mucus secretion from cholangiocytes. ACh also activates neurons, triggering neurogenic inflammation or organ-specific reflexes [66,67].

In taste buds, activated taste bud cells release ATP as a transmitter onto afferent gustatory neurons, transmitting signals to the brain [68,69]. ATP released by epithelial cells increases ciliary beat frequency in the nasal cavity [70]. However, it remains unclear whether tuft cells, beyond taste bud cells, release ATP in response to pathogenic or allergic stimuli.

### Tuft cell signal transduction pathways

Tuft cells can sense diverse environmental chemical signals and, in response, release various effector molecules. Upon activation of surface receptors, tuft cells employ intracellular signal transduction pathways to amplify and propagate these signals, and the resulting intracellular signaling networks ultimately dictate the release of specific effector molecules onto adjacent immune cells, epithelial cells, and/or nerve terminals (Figure 2). Tuft cells express numerous GPCRs, whose activation triggers G protein- or arrestin-mediated signaling cascades. Tuft cells have been shown to express canonical taste signaling proteins, including the G protein α subunit Gα-gustducin, G protein Gβγ subunits Gβ1, Gβ3, and Gγ13, phospholipase β2 (PLCβ2), inositol 1,4,5-trisphosphate receptor 2 (IP3R2) and Trpm5. Ligand binding to a GPCR induces a conformational change that activates the heterotrimeric G protein, which dissociates into Gα and Gβγ moieties, each of which engages downstream effectors, including phospholipases, adenylate cyclases (ACs), protein kinases, and ion channels. As a member of the Gαi subfamily, Gα-gustducin inhibits AC activity but does not activate PLCβ2. Instead, Gβ1γ13 or Gβ3γ13 stimulates PLCβ2, generating the second messengers diacyl glycerol (DAG) and inositol-1,4,5-triphosphate (IP3). IP3 binds to its channel receptor IP3R, releasing Ca^2+^ from intracellular stores such as the endoplasmic reticulum. Elevated cytoplasmic Ca^2+^ activates the nonselective monovalent ion channel Trpm5, causing Na^+^ influx, membrane depolarization, and release of effectors such as IL-25 and ACh.

Notably, both Tas2r bitter taste receptors in taste bud cells and the succinate receptor Suncr1 in tuft cells utilize this Gα-gustducin-Gβγ13-PLCβ2-IP3R-Trpm5-IL-25 pathway, as does the anaerobic pathobiont microbe *Ruminococcus gnavus* via Tas2r-Gβγ13-PLCβ2-IP3R-Trpm5-IL-25 to drive tuft cell expansion in the proximal colon [23,45]. The protozoan *Tritrichomonas muris*-induced tuft cell hyperplasia is dependent on both Gα-gustducin and Trpm5, whereas the parasitic helminth *Trichinella spiralis*-evoked type 2 innate immunity requires Gβγ13 and Trpm5 [4,42]. Clearance of the helminths *Heligmosomoides polygyrus* and *Trichinella spiralis* requires Trpm5, but Gα-gustducin is dispensable for the response to *Nippostrongylus brasiliensis* [23,42]. This suggests that alternative Gα subunits, such as Gα14, may substitute for Gα-gustducin, as observed in sweet taste signaling in taste buds. Alternatively, *N. brasiliensis* and other parasites may activate multiple signaling pathways in tuft cells and/or in immune cells, with Gα-gustducin ablation obscured by redundant mechanisms. In the gallbladder, activation of free fatty acid receptor 2 (Ffar2) by propionate engages multiple pathways, culminating in Trpm5-dependent release of CysLTs and ACh from tuft cells [63].

Intracellular signaling networks exhibit greater complexity than previously assumed. For instance, Gβγ13 moieties dissociated from Gα-gustducin are unlikely to directly activate phospholipases until Gα relieves their autoinhibition [71]. Whether this mechanism operates in tuft cells remains unclear. Additionally, persistent activation of tuft cell GPCRs may trigger multiple negative feedback mechanisms, including receptor phosphorylation, internalization, and G protein activity regulation by the regulators of G protein signaling (RGS), such as RGS2, RGS9, RGS13, RGS14, RGS16, RGS19, and RGS22, which are expressed in tuft cells [14]. The specific G protein-mediated pathways regulated by these RGS proteins require further elucidation. Moreover, the roles of certain second messengers, such as DAG, remain incompletely characterized, particularly their potential involvement in epigenetic modifications of tuft cell chromatin, which may encode and memorize cellular responses to pathogens.

### Tuft cells and inflammation resolution

The primary function of tuft cells is associated with innate immunity, encompassing the stimulation of group 2 innate lymphoid cells, expulsion of helminths and protozoa, secretion of antimicrobial peptides, and facilitation of mucociliary clearance. The heterogeneity of tuft cells in responses to diverse environmental stimuli suggests that their roles vary across different stages of disease progression and resolution.

Tuft cells not only contribute to initial innate immune responses against pathogens but also exhibit anti-inflammatory properties. For instance, succinate-induced tuft cell expansion suppresses ileal inflammation [72], whereas the absence of Gγ13 or Trpm5 reduces expression of gasdermins C2, C3, and C4, leading to increased epithelial cell death and colonic tissue damage in murine models [45]. Similarly, patients with inflammatory intestinal diseases exhibit reduced tuft cell populations [73]. In severely injured lungs, ectopic tuft cells emerge approximately 12 days post-H1N1 infection, indicating a limited role in the initial response or viral clearance, which typically concludes within 10 days [30]. A subset of these ectopic tuft cells expresses canonical taste signaling proteins, including Gα-gustducin, Gγ13, PLCβ2, and Trpm5, and responds to bitter tasting compounds, such as denatonium and quinine, by elevating intracellular calcium levels, a process inhibited by the inhibitors for Gβγ subunits or PLCβ2. Conditional nullification of Gγ13 expression in a subset of ectopic tuft cells in severely injured lung reduces their overall numbers, with the remaining tuft cells predominantly Gγ13-negative [37]. In the mice with a much-reduced number of Gγ13-positive ectopic tuft cells, the outcomes include more extensive lung damage, increased immune cell infiltration, pulmonary epithelial leakage, elevated pyroptosis and cell death, prolonged inflammation, greater body weight loss, delayed recovery, exacerbated fibrosis, and increased mortality.

The ablation of Gγ13-positive dysplastic tuft cells exacerbates symptoms, suggesting that these cells, in contrast to Gγ13-negative tuft cells, may play opposing roles: the former promote inflammation resolution, while the latter exhibit proinflammatory effect. Certain tuft cells produce proinflammatory cytokines, such as IL-25 and eicosanoids. On the other hand, other ectopic pulmonary tuft cells express genes encoding enzymes that synthesize specialized pro-resolving mediators (SPMs), including *Alox5*, *Alox12e*, *Alox15*, *Ptgs2*, which encodes 5-lipoxygenase (5-LOX), arachidonate 12-lipoxygenase (12-LOX), arachidonate 15-lipoxygenase (15-LOX), and prostaglandin-endoperoxide synthase II (Cox-2), respectively (Figure 2). These enzymes catalyze membrane lipids to produce SPMs, such as lipoxins, resolvins, protectins, and maresins, which limit immune cell infiltration, enhance phagocytosis to clear dead cells and debris, reduce proinflammatory cytokine and lipid mediator production, increase anti-inflammatory mediator and cytokine synthesis, and promote tissue repair and reconstitution. Maresin 1 and resolvin D1 have been detected in the lung post-viral or bacterial infection [74,75]. Collectively, these findings suggest that the Gγ13-positive and Gγ13-negative tuft cells co-ordinately regulate the later stages of disease progression, balancing inflammation resolution to accelerate recovery while maintaining limited inflammation to prevent recurrence.

## Conclusions

Recent advances in tuft cell research have elucidated key functions of these rare epithelial cells. Further investigation is needed to comprehensively understand their capabilities. Identifying the interactions between tuft cell receptors and ligands from pathogens, metabolites, and other environmental stimuli is essential to characterize the responsiveness of distinct tuft cell subtypes. Additionally, fully delineating intracellular signaling networks will enable prediction of output signaling molecules. Studies on epigenetic imprinting could enhance predictions of robust responses to the subsequent exposures to identical pathogens or stimuli. Moreover, comprehensive research is required to determine the prevalence and regulation of dedifferentiation and transdifferentiation in tuft cells.

**Perspectives**

- Tuft cells, a rare epithelial cell type, are present in various tissues, detect and respond to diverse pathogens including microbes and helminths. They play crucial roles in innate immune response, inflammation resolution, neuroimmune interactions, and tumorigenesis.
- Tuft cells employ G protein-coupled receptors, such as the succinate receptor and chemosensory receptors, to sense invading pathogens or dysbiotic microbiomes. This sensing initiates intracellular signal transduction, which triggers epigenetic regulation of gene expression and the release of effector molecules that act on adjacent cells.
- A comprehensive understanding of tuft cell heterogeneity, along with delineation of the ‘ligand-receptor-intracellular signaling networks-epigenetic modification/effector release’ axis, could enable prediction of tuft cell behaviors across different tissues—or even within the same tissue—including regulation of inflammation, dedifferentiation, and transdifferentiation.

## Open Access

This article has been published open access under our Subscribe to Open programme, made possible through the support of our subscribing institutions, learn more here: https://portlandpress.com/pages/open_access_options_and_prices#conditional

## Abbreviations

BMP: bone morphogenetic protein
COX1: cyclooxygenase 1
ChAT: choline acetyltransferase
CysLT: cysteinyl leukotriene
GPCR: G protein-coupled receptor
HDAC3: histone deacetylase 3
HPGDS: hematopoietic prostaglandin D synthase
IL-25: interleukin-25
ILC2: group 2 innate lymphoid cell
Pou2f3: Pou class 2 homeobox 3
SPM: specialized pro-resolving mediator
Trpm5: transient receptor potential cation channel subfamily M member 5
